# Pharmacokinetic Behaviour of Enrofloxacin after Single Intramuscular Dosage in American Black Vultures (*Coragyps atratus*)

**DOI:** 10.3390/antibiotics10080957

**Published:** 2021-08-09

**Authors:** Samanta Waxman, José Julio de Lucas, Guillermo Wiemeyer, Laura Torres Bianchini, Manuel Ignacio San Andrés, Casilda Rodríguez

**Affiliations:** 1 Facultad de Ciencias Veterinarias, Universidad de Buenos Aires, Chorroarin 280, Buenos Aires 1427, Argentina; gwiemeyer@fvet.uba.ar (G.W.); laura.wallaby@gmail.com (L.T.B.); 2 Consejo Nacional de Investigaciones Científicas y Técnicas (CONICET), Buenos Aires 1425, Argentina; 3 Department of Pharmacology and Toxicology, Veterinary Faculty, Universidad Complutense de Madrid, Av. Puerta de Hierro s/n, 28040 Madrid, Spain; delucas@vet.ucm.es (J.J.d.L.); misanand@vet.ucm.es (M.I.S.A.); rodfermc@vet.ucm.es (C.R.); 4 Jardín Zoológico de la Ciudad de Buenos Aires, R. de la India 3000, Buenos Aires 1425, Argentina; 5 Fundación Caburé-í, Sucre 2842, Buenos Aires 1428, Argentina

**Keywords:** black vultures, enrofloxacin, pharmacokinetic, PK/PD, Monte Carlo simulation

## Abstract

The aim of the study was to investigate the intramuscular pharmacokinetics of enrofloxacin in black vultures (*Coragyps atratus*). The pharmacokinetics of a single intramuscular dose (10 mg/kg) of enrofloxacin was studied in six vultures. Plasma concentrations of enrofloxacin and its active metabolite, ciprofloxacin, were determined by high-performance liquid chromatography (HPLCuv). Pharmacokinetic parameters were estimated using non-compartmental and compartmental analysis. After intramuscular administration, enrofloxacin showed a rapid and complete absorption, reaching a Cmax value of 3.26 ± 0.23 μg/mL at 1.75 ± 0.53 h. A long terminal half-life of 19.58 h has been observed. Using previously published MIC values to perform a PK/PD analysis, cumulative fraction responses obtained after Monte Carlo simulation for AUC/MIC > 30, 50 and 125 were 72.93%, 72.34% and 30.86% for *E. coli* and 89.29%, 88.89% and 58.57% for *Mycoplasma synoviae*, respectively. Cumulative fraction responses obtained for Cmax/MIC index were 33.93% and 40.18% for *E. coli* and *M. synoviae*, respectively. The intramuscular administration of 10 mg/kg could be appropriate to treat infectious diseases caused by gram-positive bacteria with MIC value lower than 1 µg/mL; however, although enrofloxacin showed a slow elimination in black vultures, plasma concentrations were insufficient to reach the gram-negative stablished breakpoints.

## 1. Introduction

Black vultures, as obligate scavengers, are of great value for their ecosystems by removing carrion and facilitating the flow within the food chain. This species is distributed along the American continent, being widely represented, not only in South and Central America, but also in the south of North America [1,2]. Infectious diseases are a common issue in these New World vultures; they are susceptible to *Pasteurella* *multocida* and sepsis involving other gram-negative bacteria, for which a broad-spectrum therapy with, for example, enrofloxacin, is indicated [3,4]. This drug is a fluoroquinolone with concentration-dependent bactericidal effect, good activity against gram-negative organisms, some activity against gram-positive organisms, minimal activity against anaerobic bacteria, but occasional activity against *Chlamydia* spp., *Mycoplasma* spp., and *Mycobacterium* spp. Enrofloxacin may be considered a good choice in cases of pododermatitis, showing evidence of persistent or deep infection [5]. This drug is very frequently used in the treatment of infectious diseases of vultures admitted in wildlife rehabilitation centres by the intramuscular (im) route. This route could represent an interesting alternative to oral administration, in as much as anorexia, regurgitation and emesis have been observed in these species frequently after oral administration of enrofloxacin [6]. Different pharmacokinetic behaviour has been observed in vultures compared to other birds due to a slow elimination of drugs, such as fluroquinolones [7,8] or diclofenac [9]; however, empirical dosage, recommended for other species, is used for black vultures [10]. Dose extrapolation by linear, metabolic, or allometric scaling has limitations and, ideally, dosage recommendations should be based on species-specific pharmacokinetic and pharmacodynamic studies [11,12]. Intramuscular administration of enrofloxacin is a practical route of administration, however, studies reporting the intramuscular behaviour of enrofloxacin in birds of prey are scarce [13].

Currently, fluoroquinolones are included into highest priority critically important antimicrobials in human medicine, since they are one of few available therapies for serious Salmonella spp. and *E. coli* infections [14]. Additionally, they are included in Category B (Restrict) of the categorization of antibiotic used in animals. They should be considered only when there are no antibiotics in Categories Caution or Prudence (C or D) that could be clinically effective and their use should be based on antimicrobial susceptibility testing, whenever possible [15].

For these reasons, a rational use of this drug, based on pharmacological data is necessary. The aim of our study was to investigate the intramuscular pharmacokinetics of enrofloxacin in black vultures (*Coragyps atratus*) and to perform a PK/PD analysis by Monte Carlo simulation using previously published MIC values against isolates obtained from avian species, in order to evaluate the probability of a successful clinical outcome for infections caused by such microorganisms.

## 2. Results

**Figure 1:**
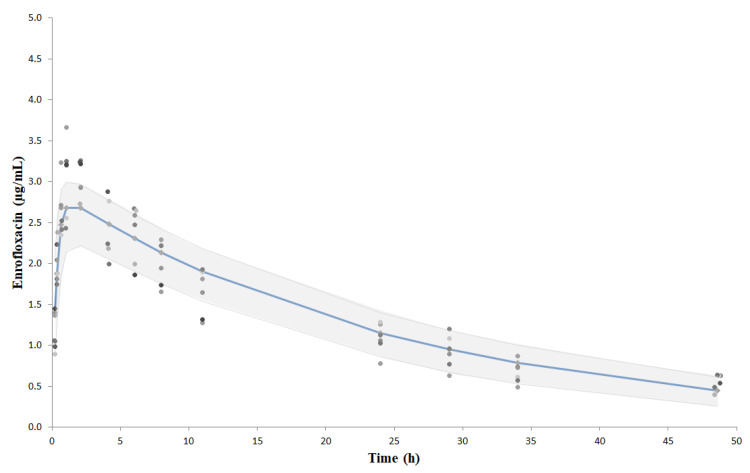
Plasma concentrations vs time profiles of enrofloxacin after a single intramuscular dose of 10 mg/kg in *Coragyps atratus* (*n* = 6). Solid lines show the final model fits. Shaded grey represents the confidence intervals of 95% of the predictive check for model evaluation after Monte Carlo simulation. Observed values of each bird are shown in greyscale.

The individual concentration vs time curves, the final model fits, and the confidence limits (95%) of the predictive check for model evaluation are shown in Figure 1. Measures of central tendency and variability of the pharmacokinetic parameters obtained after non-compartmental and compartmental analysis are shown in Table 1.

Data were best fitted to a monocompartmental model. Monte Carlo simulation of target attainment for a simulated black vulture population after intramuscular administration of enrofloxacin (10 mg/kg) is shown in Table 2 for AUC/MIC ≥ 30, 50 and 125 and Cmax/MIC ≥10 using Escherichia coli and Mycoplasma synoviae MIC values, respectively. Cumulative fraction responses (CFR) obtained after Monte Carlo simulation for AUC/MIC > 30, 50 and 125 were 72.93%, 72.34% and 30.86% for *E. coli* and 89.29%, 88.89% and 58.58% for M. synoviae, respectively. Cumulative fraction responses obtained for Cmax/MIC index were 33.93% and 40.18% for *E. coli* and M. synoviae, respectively.

## 3. Discussion

After intramuscular administration, enrofloxacin showed a rapid and complete absorption, reaching high Cmax and AUC values. Taking into account AUC values from previously published data on intravenously administered enrofloxacin to black vultures [8], a bioavailability value of 90% is estimated. This finding is in agreement with values described in other avian species, which oscillated between 90% and 99% [13,18,19,20,21]. The lowest bioavailability was observed in red-tailed hawk [13], rheas [22], and southern crested caracaras [23]. Very low values of ciprofloxacin plasma concentrations were found in black vultures. After intramuscular administration, ciprofloxacin was detected in only three birds, and only one showed detectable values between 2 to 8 h, which oscillated between 0.051 to 0.064 µg/mL. For these reasons, pharmacokinetic analysis of the active metabolite was not performed.

Compared to other species, black vultures present the highest AUC. The obtained AUC value for vultures is higher to that found for owls (15 mg/kg = 65.3 µg h/mL) [13], but almost two folds those found in houbara bustard or chicken [18,19]. The lowest AUC was found in ratites (ostriches 5 mg/kg = 1 µg h/mL, rheas, 15 mg/kg = 4.18 µg h/mL, ostriches 15 mg/kg = 6.24 µg h/mL) [21,22,24]. If AUC is corrected by the administered dose (AUC/D), in general, birds of prey show high values, black vultures showing the highest one (vultures 7.39; great horned owls 4.35, red tailed hawks 3.60; caracaras 3.44 kg h/L), while ratites show very low values [22,23,24]. These findings could be related to a slow enrofloxacin clearance and a low extraction ratio observed in vultures after intravenous administration [8]. This behaviour could also be related to a long enrofloxacin permanence in black vultures, since elimination half-life showed values higher than 18 h. Compared to other avian species, we observed that vultures present the longest body permanence, with an elimination half-life almost double that observed in chickens (10.6 h) [19], and is almost 20 times longer than in ostriches [21].

These high Cmax and AUC values could be of clinical relevance for a concentration dependent-antimicrobial as enrofloxacin. However, when the probability analysis using a Monte Carlo simulation and previously published pharmacodynamic values is performed, the pharmacokinetic profile does not seem to be enough to reach a clinical outcome. Most experts agree that a Cmax/MIC ratio around 8 to 10 or an AUC/MIC ratio greater than 100 or 125 are associated with clinical success. For this reason, those breakpoints have been taken into account in our study. The probability of target attainment (PTA) findings revealed that the empirical enrofloxacin dose could be appropriate for patients with *E. coli* or *M. synoviae* infections with MIC values of 0.125 or 0.25, respectively, when a target cut-off of AUC/MIC > 125 and Cmax/MIC > 10 is to be achieved. However, it has been stated that infections caused by gram-positive bacteria can be successfully treated even when AUC/MIC ratios are lower than 100. Thus, for this kind of infections, a ratio of 50 has been proposed as acceptable [25]. Moreover, Ambrose et al. (2001) [26] have shown that a successful clinical outcome can occur even at AUC/MIC ratios around 30 for community-acquired pneumococcal pneumonia. If 30 and 50 cut-off targets were used, PTA > 90% can be obtained for MIC values ≤ 1 μg/mL.

The cumulative fraction of response pertains to the success probability for a treatment without clinical susceptibility of the isolated pathogen. Data from the assessment of CFR (%) for the enrofloxacin dose evaluated in black vultures show that CFR reached <90% for *E. coli* and *M. synoviae* at an AUC/MIC of 125 h. The obtained CFR values are very low, barely exceeding 50% for this breakpoint, although this species has shown a slower enrofloxacin elimination and higher Cmax and AUC values than those found in all the previously studied avian species. The estimated values have been slightly better for stablished breakpoints of 30 and 50; especially to *M. synoviae*, being very close to 90%.

## 4. Materials and Methods

The experiment was performed in adult healthy black vultures (*Coragyps atratus*, *n* = 6), weighing 1.8–2.2 kg, housed at Buenos Aires Zoological Garden, Argentina. Complete physical examination, haematological analysis, maintenance of body weight and routine acceptance of daily meals were used as criteria for selection of healthy animals. No drugs were administered for at least two months prior to the start of the experiment. Vultures were housed in captivity, fed in an appropriate manner for the species, and had access to water ad libitum.

A commercial 5% enrofloxacin injectable solution (Baytril, Bayer, Argentina) was used. Ofloxacin, enrofloxacin and ciprofloxacin analytical standards were purchased from Sigma-Aldrich (Sigma-Aldrich, Madrid, Spain). Stock standard solutions were prepared from the reference standards, dissolved in 0.1 N formic acid in water, and stored at −80 °C. Ofloxacin (Sigma-Aldrich, Madrid, Spain) was used as internal standard.

A single 10 mg/kg im administration of enrofloxacin was performed through a 24 G catheter placed in the left ulnar vein. Blood samples (0.6 mL at each time point) were collected from the medial tarsal vein with a 27 G needle attached to a 1 mL heparinized syringe at 0, 10, 15, 35 min, 1, 2, 4, 6, 8, 11, 24, 29, 34, and 48 h after im administration. For each bird, the total sample volume did not exceed 10% of the blood volume of the animal. Plasma was separated immediately in a refrigerated centrifuge and frozen at −80 °C until analysed.

Sample processing and drug detection methodologies for both enrofloxacin and ciprofloxacin were slightly modified from a previously published method [23]. Enrofloxacin was quantified using high performance liquid chromatography (HPLC/u.v.: Spectra SystemThermo Separation Products Inc., Madrid, Spain) where the separation was accomplished using an ion-pairing reverse-phase column (PR C-18 5 µm 150 × 4.6 mm). No chromatography interferences were observed in the retention time of the analytes. The limit of quantification (LOQ) was 0.025 µg/mL for enrofloxacin and 0.05 µg/mL for ciprofloxacin, and the calibration curve was linear up to 5 µg/mL (R2 > 0.99 for both drugs). LOQ were determined by the lowest point on a linear calibration curve that was within precision and accuracy acceptance criteria. Intraday precision was <8% and inter-day precision was <12%. Accuracy ranged between 82–120% and 88–113%, for enrofloxacin and ciprofloxacin, respectively.

Plasma concentrations of enrofloxacin after im administration were subjected to compartmental and non-compartmental analysis using the software package PCnonlinV4.0 (Statistical Consultants Inc., Lexintong, MA, USA). The non-compartmental pharmacokinetic parameters, determined for each individual animal, were: the observed Cmax, Tmax, Clast and Tlast; area under the plasma concentration vs time curve (AUC) [AUC values were calculated using trapezoidal rule from time 0 to the last concentration time point (AUCt)], mean residence time [MRT, where MRT = AUMC/AUC; calculated from time 0 to the last concentration time point (MRTt)], elimination rate constant (λ, calculated as the slope of the terminal phase of the plasma concentration curve that included a minimum of four points) and terminal half-life (t1/2λ, where t1/2λ = 0.693/λ). As the percentage ratio 100 × AUCt/AUCinf exceeds 80% in each subject, these AUCinf values were not considered for the analysis [27]. The monocompartmental parameters were: Estimated Cmax and Tmax; AUC calculated to infinite (AUCinf), absorption (T_1/2K01_) and elimination (T_1/2K10_) half-lives.

Pharmacokinetic concentration-time profiles of enrofloxacin administered by the im route were plotted under single dose condition by Monte Carlo simulation, based on the descriptive statistical data obtained for pharmacokinetic parameters (subjects = 100). AUC and Cmax parameters obtained from this simulated curves and previously published MIC values from *E. coli* strains [16] and *M. synoviae* [17] were used to perform a PK/PD analysis based on Monte Carlo simulation (subjects = 10,000). A log-normal distribution was assumed for PK parameters. Monte Carlo simulations were conducted using Oracle Crystal 174 Ball V.11.1.1.0.00 software (Oracle Corporation, Redwood Shores, CA, USA). The PK/PD index and pharmacodynamic target associated with the efficacy of enrofloxacin were Cmax/MIC > 10 and AUC/MIC > 30, 50 and 125 [28]. The probability of target attainment, defined as the probability of the dose regimen to achieve a determined PK/PD endpoint for each MIC value and the probability of a dose regimen to achieve a determined PK/PD endpoint taking into account the entire MIC distribution of the tested bacterial population (cumulative fraction of response; CFR), was calculated for the proposed dose regimen of enrofloxacin. PTA and CFR values > 90% were considered adequate [29].

## 5. Conclusions

In conclusion, the im enrofloxacin administration of 10 mg/kg in *Coragyps atratus* could be appropriate to treat infectious diseases caused by gram-positive bacteria with MIC values lower than 1 µg/mL. However, although enrofloxacin showed a slow elimination in black vultures, the observed plasma concentrations were insufficient to reach the gram-negative stablished breakpoints. However, it should be taken into account that, as there is no data available for birds of prey, the calculations were performed using MIC values against poultry isolates, which are probably higher than those corresponding to wild birds’ populations. Further studies, evaluating higher doses and its adverse reactions, should be performed.

## Figures and Tables

**Table 1:** Pharmacokinetic parameters obtained after enrofloxacin intramuscular administration (10 mg/kg) in black vultures (*Coragyps atratus*) (*n* = 6).

Pharmacokinetic Parameter	Mean	Median	Geometric Mean	Standard Deviation	Range	CV (%)
Non-Compartmental						
T_max_ (h)	1.75	2.08	1.67	0.53	1.05	30.3
C_max_ (µg/mL)	3.26	3.25	3.25	0.23	0.73	7.1
T_1/2__λ_ (h) ^1^	19.58	19.51	19.45	1.74	5.32	8.9
AUC_t_ (µg·h/mL)	60.41	63.39	60.00	7.49	17.89	12.4
MRT_t_ (h) ^1^	17.00	16.97	16.96	0.96	2.90	5.6
Tlast (h)	48.62	48.66	48.62	0.20	0.45	0.4
Clast (µg/mL)	0.55	0.58	0.55	0.10	0.24	18.2
Monocompartmental						
AUC_inf_ (µg·h/mL)	75.79	80.44	75.05	11.2	26.97	14.8
T_1/2K01_ (h) ^1^	0.24	0.25	0.23	0.07	0.18	29.2
T_1/2K10_ (h) ^1^	18.16	18.56	17.99	1.86	4.96	10.2
T_max_ (h)	1.53	1.62	1.49	0.37	0.9	24.2
C_max_ (µg/mL)	2.72	2.72	2.72	0.18	0.49	6.61

^1^ Harmonic mean.

**Table 2:** Monte Carlo simulation (*n* = 10000 subjects) of target attainment for a simulated black vulture population after intramuscular administration of enrofloxacin at 10 mg/kg for MIC distribution of *E. coli* and *M. synoviae* previously published [16,17].

**PK/PD Index**		**PTA (%) with an *E. coli* MIC**											
		0.008	0.016	0.03	0.06	0.125	0.25	0.5	1	2	4	8	16
AUC/MIC = 125		100	100	100	100	100	50.12	0.01	0	0	0	0	0
	CFR (%)	30.86											
AUC/MIC = 50		100	100	100	100	100	100	100	100	0.15	0	0	0
	CFR (%)	72.34											
AUC/MIC = 30		100	100	100	100	100	100	100	100	69.71	0	0	0
	CFR (%)	72.93											
Cmax/MIC > 10		100	100	100	100	100	94.31	0	0	0	0	0	0
	CFR (%)	33.93											
		**PTA (%) with an *M. synoviae* MIC**											
		0.1	0.25	0.5	1	2.5	10						
AUC/MIC = 125		100	100	50.87	0	0	0						
	CFR (%)	58.57											
AUC/MIC = 50		100	100	100	100	0	0						
	CFR (%)	88.89											
AUC/MIC = 30		100	100	100	100	7.27	0						
	CFR (%)	89.29											
Cmax/MIC > 10		100	87.2	0	0	0	0						
	CFR (%)	40.18											

## Data Availability

The data presented in this study are available on request from the corresponding author.

## References

[1] The IUCN Red List of Threatened Species. www.iucnredlist.org.

[2] Holland A.E., Byrne M.E., Bryan A.L., DeVault T.L., Rhodes O.E., Beasley J.C. (2017). Fine-scale assessment of home ranges and activity patterns for resident black vultures (*Coragyps atratus*) and turkey vultures (*Cathartes aura*). PLoS ONE.

[3] Graham J.E., Heatley J.J. (2007). Emergency Care of Raptors. Vet. Clin. N. Am. Exot. Anim. Pract..

[4] Summa N.M., Sanchez-Migallon Guzman D. (2017). Evidence-Based Advances in Avian Medicine. Vet. Clin. N. Am. Exot. Anim. Pract..

[5] Blair J., Bumblefoot A. (2013). Comparison of Clinical Presentation and Treatment of Pododermatitis in Rabbits, Rodents, and Birds. Vet. Clin. N. Am. Exot. Anim. Pract..

[6] Stout J.D. (2016). Common Emergencies in Pet Birds. Vet. Clin. N. Am. Exot. Anim. Pract..

[7] García-Montijano M., Waxman S., de Lucas J.J., Luaces I., San Andrés M.D., Rodríguez C. (2011). Disposition of marbofloxacin in vulture (*Gyps fulvus*) after intravenous administration of a single dose. Res. Vet. Sci..

[8] Waxman S., Prados A.P., de Lucas J.J., Wiemeyer G., Torres-Bianchini L., San Andrés M.I. (2019). Evaluation of allometric scaling as a tool for extrapolation of the enrofloxacin dose in American black vultures (*Coragyps atratus*). Am. J. Vet. Res..

[9] Naidoo V., Wolter K., Cuthbert R., Duncan N. (2009). Veterinary diclofenac threatens Africa’s endangered vulture species. Regul. Toxicol. Pharmacol..

[10] Hawkins M.G., Sanchez-Migallon Guzman D., Beaufrêre H., Lennox A., Carpenter J.W., Carpenter J.W. (2018). Antimicrobial Agents Used in Birds. Exotic Animal Formulary.

[11] Hunter R.P., Mahmood I., Martinez M.N. (2008). Prediction of xenobiotic clearance in avian species using mammalian or avian data: How accurate is the prediction?. J. Vet. Pharmacol. Therap..

[12] Hunter R.P., Isaza R. (2008). Concepts and issues with interspecies scaling in zoological pharmacology. J. Zoo Wildl. Med..

[13] Harrestein L.A., Tell L.A., Vulliet R., Needham M., Brandt C.M., Brondos A., Stedman B., Kass P.H. (2000). Disposition of enrofloxacin in red-tailed hawks (*Buteo jamaicensis*) and great horned owls (*Bubo virginianus*) after a single oral, intramuscular or intravenous dose. J. Avian Med. Surg..

[14] (2018). Critically Important Antimicrobials for Human Medicine 6th Revision. https://apps.who.int/iris/bitstream/handle/10665/312266/9789241515528-eng.pdf.

[15] Categorization of Antibiotics Used in Animals Promotes Responsible Use to Protect Public and Animal Health EMA/688114/2020. https://www.ema.europa.eu/en/news/categorisation-antibiotics-used-animals-promotes-responsible-use-protect-public-animal-health.

[16] Vanni M., Meucci V., Tognetti R., Cagnardi P., Montesissa C., Piccirillo A., Rossi A.M., Di Bello D., Intorre L. (2014). Fluoroquinolone resistance and molecular characterization of gyrA and parC quinolone resistance-determining regions in *Escherichia coli* isolated from poultry. Poult. Sci..

[17] Gerchman I., Lysnyansky I., Perk S., Levisohn S. (2008). In vitro susceptibilities to fluoroquinolones in current and archived *Mycoplasma gallisepticum* and *Mycoplasma synoviae* isolates from meat-type turkeys. Vet. Microbiol..

[18] Abd el-Aziz M.I., Aziz M.A., Soliman F.A. (1997). Pharmacokinetic evaluation of enrofloxacin in chickens. Br. Poult. Sci..

[19] Bugyei K., Black W.D., McEwen S. (1999). Pharmacokinetics of enrofloxacin given by the oral, intravenous and intramuscular routes in broiler chickens. Can. J. Vet. Res..

[20] Bailey T.A., Sheen R.S., Silvanose C., Samour J.H., Garner A., Harron D.W. (1998). Pharmacokinetics of enrofloxacin after intravenous, intramuscular and oral administration in houbara bustard (*Chlamydotis undulata macqueenii*). J. Vet. Pharmacol. Ther..

[21] de Lucas J.J., Rodriguez C., Waxman S., González F., Uriarte I., San Andrés M.I. (2004). Pharmacokinetics of enrofloxacin after single intravenous and intramuscular administration in young domestic ostrich (*Struthio camelus*). J. Vet. Pharmacol. Ther..

[22] de Lucas J.J., Navarro J.L., Rubio S., Vignolo P.E., Asis V.C., González F., Rodríguez C. (2008). Pharmacokinetic behaviour of enrofloxacin in greater rheas following a single-dose intramuscular administration. Vet. J..

[23] Waxman S., Prados A.P., de Lucas J., San Andres M.I., Sassaroli J.C., Orozco M., Argibay H., Rodriguez C. (2013). Pharmacokinetic and pharmacodynamic properties of enrofloxacin in southern crested caracaras (*Caracara plancus*). J. Avian Med. Surg..

[24] de Lucas J.J., Solano J., González F., Ballesteros C., San Andrés M.I., Martín Von Kauffmann C., Rodríguez C. (2013). Pharmacokinetics of enrofloxacin after multiple subcutaneous and intramuscular administrations in adult ostriches. Br. Poult. Sci..

[25] Drusano G.L., Johnson D.E., Rosen M., Standiford H.C. (1993). Pharmacodynamics of a fluoroquinolone antimicrobial agent in a neutropenic rat model of Pseudomonas sepsis. Antimicrob. Agents Chemother..

[26] Ambrose P.G., Grasela D.M., Grasela T.H., Passarell J., Mayer H.B., Pierce P.F. (2001). Pharmacodynamics of fluoroquinolones against Streptococcus pneumoniae in patients with community-acquired respiratory tract infections. Antimicrob. Agents Chemother..

[27] Hauschke D., Steinijans V., Pigeot I., Hoboken N.J. (2007). Bioequivalence Studies in Drug Development: Methods and Application.

[28] Papich M.G. (2014). Pharmacokinetic-pharmacodynamic (PK-PD) modeling and the rational selection of dosage regimes for the prudent use of antimicrobial drugs. Vet. Microbiol..

[29] Asín-Prieto E., Rodríguez-Gascón A., Isla A. (2015). Applications of the pharmacokinetic/pharmacodynamic (PK/PD) analysis of antimicrobial agents. J. Infect. Chemother..

